# Gender incongruence and gender dysphoria in childhood and adolescence—current insights in diagnostics, management, and follow-up

**DOI:** 10.1007/s00431-020-03906-y

**Published:** 2020-12-18

**Authors:** Hedi Claahsen - van der Grinten, Chris Verhaak, Thomas Steensma, Tim Middelberg, Joep Roeffen, Daniel Klink

**Affiliations:** 1 Department of Pediatrics, Amalia Children’s Hospital, Radboud University Medical Center, Nijmegen, Netherlands; 2 Department of Medical Psychology, Amalia Children’s Hospital, Radboud University Medical Center, Nijmegen, Netherlands; 3 Center of Expertise on Gender Dysphoria and Department of Medical Psychology, Amsterdam UMC, location VUmc, Amsterdam, Netherlands; 4 Department of plastic surgery, University Medical Centre Groningen, University of Groningen, Groningen, Netherlands; 5 Genderteam South Netherlands, Mutsaersstichting, Venlo/Eindhoven, Netherlands; 6 Division of Pediatric Endocrinology and Diabetes, ZNA Queen Paola Children’s Hospital, Antwerp, Belgium and Division of Pediatric Endocrinology, Department of Internal Medicine and Pediatrics, Ghent University Hospital and Ghent University, Ghent, Belgium

**Keywords:** Gender incongruence, Gender dysphoria, Transgender care, Pubertal suppression, Gender-affirming treatment

## Abstract

Gender incongruence (GI) is defined as a condition in which the gender identity of a person does not align with the gender assigned at birth. Awareness and more social acceptance have paved the way for early medical intervention about two decades ago and are now part of good clinical practice although much robust data is lacking. Medical and mental treatment in adolescents with GI is complex and is recommended to take place within a team of mental health professionals, psychiatrists, endocrinologists, and other healthcare providers. The somatic treatment generally consists of the use of GnRH analogues to prevent the progression of biological puberty and subsequently gender-affirming hormonal treatment to develop sex characteristics of the self-identified gender and surgical procedures. However to optimize treatment regimens, long-term follow-up and additional studies are still needed.

**Table:** 

**What is known** *• The prevalence of gender dysphoria increased significantly in the past years and can lead to significant complaints and burdens especially during puberty.* *• Pubertal suppression and gender-affirmed treatment can be effectively used in adolescence with gender dysphoria.* **What is new** *• Transgender mental and medical healthcare is a long-lasting process during which not only the child/adolescent with GI but also their parents/family have to be counseled in making choices about their social, medical, and legal transitions.* *• There are an increasing number of transgender persons defining as nonbinary. Therefore, an individualized approach by an experienced team is necessary.*

**What is known**

*• The prevalence of gender dysphoria increased significantly in the past years and can lead to significant complaints and burdens especially during puberty.*

*• Pubertal suppression and gender-affirmed treatment can be effectively used in adolescence with gender dysphoria.*

**What is new**

*• Transgender mental and medical healthcare is a long-lasting process during which not only the child/adolescent with GI but also their parents/family have to be counseled in making choices about their social, medical, and legal transitions.*

*• There are an increasing number of transgender persons defining as nonbinary. Therefore, an individualized approach by an experienced team is necessary.*

## Introduction, definition, and epidemiological data

Gender identity and gender variation are frequently discussed in today’s modern society. Less than 50 years ago, the traditional roles of men and women were clearly defined. There was a taboo around topics such as homosexuality and gender incongruence. Nowadays, most Western societies are more open to variations in sexuality and gender identity. Gender identity refers to the identification of a person of being male, female, or neither/both [1]. Gender incongruence (GI) is defined as a condition in which the gender identity of a person does not align with the gender assigned at birth.

Persons with GI who experience significant burdens are in the DSM classification described as gender dysphoria (GD).

Opposite to the binary approach of being male or female, the concept of gender becomes nowadays accepted as a continuum. Nonbinary persons are currently reported in up to 10% of the persons with GI [2, 3].

The prevalence of GD in children and adolescence is reported to be 0.6–1.7% and depends on the selection of the study cohort, age, and method of investigation [4]. In recent years, the number of children and adolescents seeking help with GI and GD has increased sharply and especially in children not all persons with GI represent with symptoms of GD [4–6]. A remarkable change concomitant with the increase in referrals is a shift in the sex ratio of clinically referred adolescents, with more birth assigned girls now referred than birth assigned boys [7].

In this review, we describe the current approach of GI/GD in children and adolescents. We address etiological factors, assessment procedures, counseling issues, and decisions on/options available for medical and surgical treatment options. Furthermore, we describe recent evidence on long-term outcome in young adulthood.

## Etiological factors

To date, the etiology of GI is still largely unidentified. Current research suggests that psychosocial and biological factors play a role in the development of GI. Indeed, there are findings that suggest there is a biological/anatomical base for GI: postmortem brain studies have shown that specific brain structures that are found to be different between men and women without GI show strong resemblance in volume and the number of neurons in people with GI to the gender one identifies [13]. Recent studies have focused on brain connectivity between people with and without GI, showing differences in brain networks related to body image [15]. However, much is still unknown about the extent and period in which psychosocial and biological factors make a (specific) contribution in the development, as well as about the possible interactions between the various factors involved [5, 8].The earlier studies on the development of GI primarily focused on the influence of individual psychological factors, such as the mother-child interaction and/or the absence or passive presence of the father [9]. The evidence for the role of individual psychological factors is however limited [10]. Later theories hypothesized that the development of GI was a multiple factor process in which parental, child, and environmental factors have their contribution. GI develops at the moment that general child factors (such as anxiety) and parental factors (psychological difficulties in the parents) as well as specific factors (such as a lack of limit setting of parents) occur simultaneously during a certain critical time frame in the development of the child [11]. Although for some general factors like anxiety in the child, research found some support, evidence for specific child and parental factors, is limited or lacking [10].

Research on the role of biological factors involved in the development of GI has mainly focused on genetic factors the role of (prenatal) sex hormones and differences in the brain. Genetic contribution in the development of GI has been demonstrated in twin studies, showing a high concordance of GI in monozygotic twin pairs and discordance of GI in dizygotic twin pairs (see for review [12]) but true candidate genes have not yet been identified [13]. Brain imaging studies have found support for the role of prenatal hormone (androgen) exposure in the development of GI. Various studies, in which various measures have been used, have shown that the brains of people with GI show resemblance to the brains of the gender they identify with and differences to the brains of the gender they were assigned with; the variations in findings are however large between studies [12].

Although for all these factors, significant contribution has been observed; in conclusion, the causative role between genes, hormones, brains structure, behavior, and GI is still questioned [16] and how these different factors relate to each other is less clear and less studied.

## Assessment procedure for children and adolescents

There is a great diversity in questions from children, adolescents, and parents regarding their gender, when they seek professional help. Some have socially transitioned at a young age and feel certain about their gender identity, while others are still exploring their gender identity even in (late) adolescence. Exploring one’s gender identity is a normal developmental process [1, 2] during which a child learns to label their own gender (gender labeling) and experience a stable gender identity (gender constancy) [14].Gender incongruence in childhood tends to be more in development and fluid than in adolescence where gender identity seems to be more fixed [6, 12, 15].

In young children, mostly parents seek help for their concerns on how to handle the gender questions of their child. In (young) adolescents on the other hand, there is a shift towards the children themselves, when their physical changes as a result of their pubertal development urge their need for support. Special attention is needed regarding the language in which the child or adolescent is addressed to. Words with a gender statement such as “boy,” “girl,” “son,” “daughter,” “he,” and “her” can be experienced as uncomfortable for both children with GI and their parents. It is important to be aware of these emotions and to take a step towards gender-sensitive work by asking how someone wants to be addressed.

The assessment procedure in children and adolescents is similar. In line with the recommendations of the World Professional Association for Transgender Health (WPATH) and Dutch quality standard for mental health in transgender care (www.richtlijnendatabase.nl): in the first consultation with the parents and their child conjointly, their specific aims are discussed. Subsequently, general diagnostic sessions are conducted with the child/adolescent and parents separately. The sessions with the child/adolescent are focused on gaining a general perspective of the psychosocial, cognitive, and emotional development and investigating beliefs regarding their gender identity—expression. Additionally, in adolescents, their psychosexual development is addressed. Diagnostic assessment is focused on family background and on both general as gender-specific dynamics. Gender dysphoria is a formal diagnosis in the DSM-5 classification and separately defined for children (302.6) and adolescents (302.85) and adults (302.6). It is defined as a difference between one’s experienced and assigned gender, and significant distress or problems in functioning lasting for at least 6 months (https://www.psychiatry.org/patients-families/gender-dysphoria/what-is-gender-dysphoria) persistent and intense distress about one’s assigned gender, manifested prior to puberty.

Psychiatric problems, such as internalizing problems, i.e., anxiety and depression, increased incidence of suicidal behaviors, and autism spectrum disorders [1, 3] are more prevalent in children and adolescents with GI [17]. Therefore, the diagnostic sessions are also aimed to address these possible coexisting problems. Internalizing problems are thought to be reactive to feelings of GI and/or in response to social stigma. Many children report a history of peer problems in terms of being bullied [4]. The cause of the relationship between autism spectrum disorders and gender dysphoria is still in debate and is suggested to be related to brain and hormonal functioning, or mentalizing ability [16, 17].

BOX 1
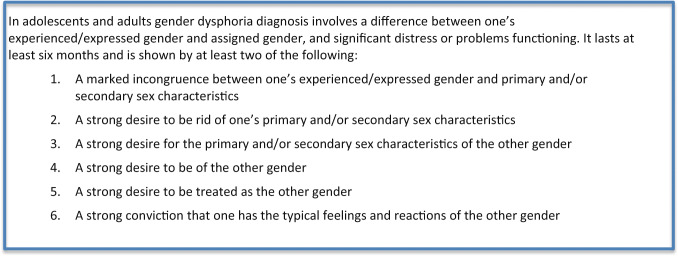


BOX 2
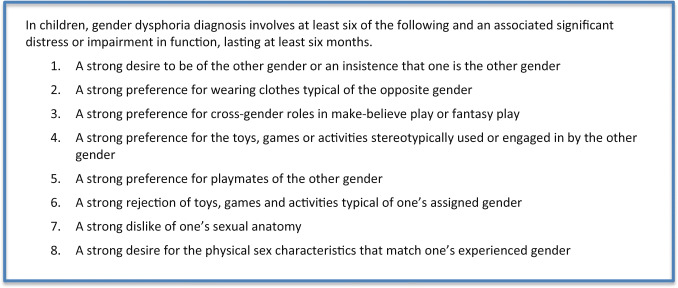


## Psychological support for children and adolescents with gender incongruence

**Fig. 1:**
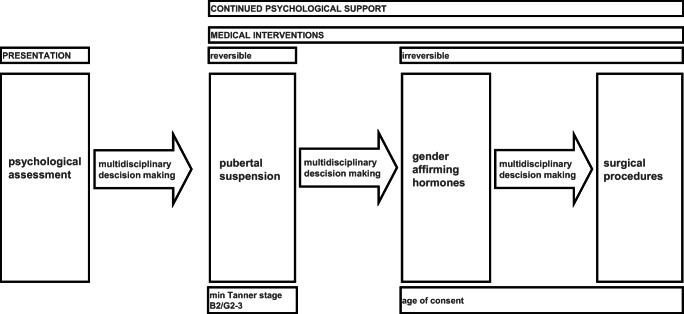
Approach towards children and adolescence with gender incongruence/dysphoria

There is no evidence-based guideline for psychological support for children and adolescents. Treatments aimed to change gender identity have not shown to be effective and now are widely considered to be unethical [WPATH SOC-7]. But still, optimal processes and outcomes of psychological interventions are under debate and range from supporting social transition to supporting feelings in line with sex assigned at birth [18]. Support focuses on psychoeducation, e.g., explaining to the parents that exploration of gender expressions is a part of a developmental process and, in the majority of the children, does not result in persistent gender dysphoria in adolescence. In children with GI finding, a balance between watchful waiting and taking steps towards gender-affirming interventions is an important goal [19]. During childhood, much attention is given to decreasing distress as a result of the gender incongruence and to preparing/supporting the child and parents in the exploration and development towards the possible steps when their endogenous puberty development commences. Transgender mental and medical healthcare is a long-lasting process during which the child/adolescent with GI and their parents are counseled in making choices about their social, medical, and legal transitions (Fig. 1).

In adolescents, medical interventions are possible and psychological counseling is aimed to guide and support the adolescent and parents during this process. After the initial diagnostic phase as described previously, possibilities for medical treatment including hormonal treatment, surgery and fertility preservation, are discussed and balanced with expectations of both the adolescent and the parents. During the phase of medical interventions, they are continuously supported until the desired medical steps are completed. Since it has been shown that peer group support is a valuable tool during medical transition [20], it is advisable to contact support groups or self-help organizations.

## Medical treatment

### Pubertal suppression

The development of their biological secondary sex characteristics is generally a highly distressful experience for adolescents with GI/GD that may lead to serious complaints of psychological functioning and behavior. Therefore, pubertal suppression (PS) has been introduced in several expert centers to prevent or stop pubertal development [21] (Fig. 1). It is well known that the use of long-acting GnRH analogous (GnRHa) to suppress gonadotropins can effectively prevent the progression of puberty. There is longstanding experience with the use of this treatment in young children with central precocious puberty (CPP) [15]. In this group, pubertal suppression is fully reversible. For adolescents with GI/GD, this treatment offers the opportunity to create more time for diagnostics and mental health evaluation. Current consensus states that pubertal suppression should not start before Tanner stage 2 (birth assigned females breast Tanner 2, birth assigned males testicular volume of 6–8 ml) to enable children to experience the changes of their own pubertal development as desistence of GI has been described during puberty. The most often used GnRH is an intramuscular injection every 3 months but also longer acting versions and also surgically implanted antagonists are available. Criteria for starting PS are (1) confirmed diagnosis GD/GI by an experienced mental health professional (see previous section), (2) written informed consent, (3) minimal Tanner 2, and (4) preferably, there is sufficient parental support and no interfering health issues. In the decision to treat, the presence of additional risk factors/interfering health issues has to be balanced with the possibility that withholding treatment will cause harm to the patient.

After starting treatment, some secondary sex characteristic which had already developed may decrease such breast size and testes volume. Also, a withdrawal bleeding may occur in individuals with GI who were assigned female at birth.

GnRHa is generally well tolerated with the exception of hot flushes early in treatment and local reactions such as redness and pain [22, 23]. Furthermore, emotional lability and mood changes are described [24]. Hitherto, only arterial hypertension was reported as an adverse event in transgender youth in 3 cases in a cohort of 138 subjects. Hypertension was reversible upon cessation of triptorelin [22, 25–27].

In early puberty, epiphyseal plates are still open, and height gain and final height may be influenced. Since little is known on growth patterns during and after treatment, the patient has to be counseled about the possible effects on growth but cannot be provided with based data on height gain and final height.

Despite the positive effect on pubertal suppression, there is still an ongoing debate on early medical intervention [26]*.* Opponents suggested that especially in the younger group, an unstable pattern of gender variations exists with reversible gender incongruence in most of the children [26]. Until now, there is limited data about the effect of pubertal suppression on mental health, all showing an improvement of mental health and quality of life [10, 28–31]. However, long-term outcome studies are necessary to confirm these results in larger cohorts.

### Gender-affirming hormonal treatment

Subsequent to GnRHa treatment, synthetic sex steroids are added to induce the development of sexual characteristics of the identified gender. There are generally two treatment regimes. When GnRHa treatment had started in an early Tanner stage, the “new” puberty is induced with a dosage scheme that is also common in prepubertal hypogonadal adolescents. Alternatively, when GnRHa treatment had started in physically matured persons and the duration of the hypogonadal state was limited, hormones can be started at a higher dose and more rapidly increased. An additional advantage of GnRHa treatment is that hormones do not have to be administered in supraphysiological dosages, which would otherwise be needed to suppress endogenous sex steroid production [32]. An example of pubertal induction is given in Table 1.

**Table 1:** Example of puberty induction and follow-up protocol in adolescents with gender dysphoria

	Birth assigned females	Birth assigned males
Treatment		
	Testosterone esters (im, sc)	17β estradiol
Increasing dose every 6 months	25 mg/m^2^/2 wks 50 mg/m^2^/2 wks 75 mg/m^2^/2 wks 100 mg/m^2^/2 wks	5 ucg/kg/day 10 ucg/kg/day 15 ucg/kg/day 20 ucg/kg/day
Adult dose	100–250 mg every 2–3 wks	2–6 mg/day
Follow-up		
Every 3–6 months	Height, weight, blood pressure, Tanner stage	
Every 6–12 months	Hemoglobin/hematocrit, lipids, testosterone, vitamin D (25OH)	Prolactin, estradiol, vitamin D (25 OH)
Every 1–2 years	BMD (DEXA scan), bone age (if indicated)	

IM, intramuscularly; SC, subcutaneously, BMD, bone mineral density; adapted from [26]

#### Individuals with GI who were assigned male at birth

The natural 17-beta estradiol is preferred over synthetic estrogens which have a more thrombogenic profile. For a pubertal induction, the recommended start dosage is 5 mcg/kg/day, followed by 6 monthly increments of 5 mcg/kg until a maintenance dosage of 2–4 mg is reached. In transgender girls who commenced GnRHa when in Tanner stage 4/5, estrogens can be given at a daily start dosage of 1 mg after a period of gonadal suppression varying from 3 to 6 months. This dosage can be increased to 2 mg after 6 months. Feminization includes breast development, generally starting within 3 months after start treatment, and body shape alteration with an increase in hip and decrease in waist circumference [33, 34]. Transgirls need gonadal suppressive treatment regardless of the estrogen dosage until gonadectomy has been performed. GnRHa are preferred over other anti-androgens such as cyproterone acetate or spironolacton. Since no data available on how efficient exogenous synthetic sex steroids can suppress the gonadal axis during puberty, GnRHa are to be continued until gonadectomy, when started in early puberty.

#### Individuals with GI who were assigned girls at birth

For pubertal induction, the use of testosterone esters injections is recommended. The start dosage is 25 mg/m^2^ every 2 weeks intramuscularly (im) and is increased with 25 mg/m^2^ every 6 months. The maintenance dosages vary from 200 mg per 2 weeks for testosterone monoesters, such as testosterone enanthate, to 250 mg im per 3–4 weeks for testosterone esters mixture. For transgender boys who started treatment in the late pubertal stage, testosterone can be started in a dosage of 75 mg im every 2 weeks, followed by the maintenance dosage after 6 months. It is advised to continue GnRHa at least until the maintenance dosage of testosterone is reached and preferred to continue until gonadectomy. With androgens, virilization of the body occurs: lowering of the voice; more muscular development, particularly in the upper body; facial and body hair growth; and clitoral growth [24, 32].

## Surgical interventions

Surgery can address the primary or secondary sex characteristics with the aim to establish greater congruence with the experienced gender. Not every transgender or gender-incongruent individual seeks surgical interventions to change sex characteristics (Table 2). The desire for an operation is for every transgender individual different. There is a great variety in the combination of the possible operations. Each person should have an individual approach to fit their surgical needs. Follow-up studies have shown a positive effect of gender-affirming surgery on postoperative outcomes such as well-being, outer appearance, and sexual function. Surgeons in the field of gender surgery usually come from different specialties, depending on the type of operation. The following surgical specialties are common in gender surgery: ENT, maxillofacial surgery, plastic surgery, urology, gynecology, and general surgery. Given the low volume within their core specialty, surgeons in the field of gender surgery need special training and should be linked closely to a specialized gender team.

**Table 2:** Overview of surgical procedures for the treatment of transgender people with GI

Birth assigned males	Birth assigned females
Breast surgery = augmentation mammoplasty with implants or lipofilling Genital surgery (sex reassignment surgery): • Penectomy = removal of penis • Orchiectomy = removal of testicles • Vulvoplasty = creation of female outer genitalia including functional neo-clitoris • Vaginoplasty = creation of female genitalia including a functional vaginal cavity using the penile and scrotal skin, creation of a functional neo-clitoris Other surgical interventions: • Facial feminization surgery (including bone structure altering surgery, rhinoplasty, blepharoplasty, forehead lift, lipofilling, use of fillers) • Liposuction or lipofilling of body fat • Voice changing surgery • Thyroid cartilage reduction • Gluteal augmentation (implants/lipofilling) • Hair reconstruction (hairline, male-type alopecia)	Breast/chest surgery: subcutaneous mastectomy, creation of a male chest and a male-type nipple/areola complex Genital surgery (sex reassignment surgery): • Hysterectomy + salpingo-oophorectomy • Urethra lengthening which can be combined with a metoidioplasty (creating a small male genitals with the use of local tissue) or with a phalloplasty (using a pedicled or free vascularized sensitive flap) • Vaginectomy • Scrotoplasty • Implantation of erection and/or testicular prostheses Other surgical interventions: • Voice surgery (rare) • Liposuction of lipofilling • Pectoral implants

Candidates for surgery should not have any significant medical and mental healthcare concerns. In preparation for surgery, it is helpful to assess the resilience of the candidate to prevent decompensation when complications occur or to help cope the persons with the efforts of post-surgery self-care. Therefore, a profound informed consent process prior to surgery is desirable.

The goal of gender-affirming surgery is to reach the appearance and function of the experienced sex characteristics and the genital appearance as “natural” as possible. However, the results in gender-affirming surgery can vary from striking and satisfying to disappointing, as complete authenticity is certainly unobtainable. Prior to surgical interventions, it is preferred that the person had lived in the self-identified gender role for a substantial period of time. The intent of this suggested sequence is to give adolescents sufficient opportunity to experience and socially adjust in their new gender role. Most gender-affirming surgical procedures are irreversible or if reversible lead to extensive scaring. These procedures should not be done if someone is under legal age to give consent for a medical procedure in a given country. Mastectomy for transgender males is considered to be less invasive procedure and can be done in people under legal age of consent to reduce gender dysphoria especially in the case extensive female breast development that cannot be hidden easily with a breast binder [35].

Most transgender boys have most often a wish for mastectomy, followed by hysterectomy. Genital operations especially the construction of a neophallus (metoidioplasty/phalloplasty) are not that often asked due to unpredictability of the outcome and complication rates of the operation [36].

In transgender girls, vaginoplasty is the mostly favored operation carried out followed by breast augmentation with implants [37].

## Long-term outcome of early medical intervention

True long-term outcome studies are currently not available but studies within a cohort of young adults at age 22 who were treated in their teens are published. With respect to bone health, it was reported that bone mass was in the normal range but not at pre-treatment level for both transgender men and transgender women. However, only in transgender women, few had T-score < −2.5 [38]. When compared to age-matched peers, young adult transgender women showed greater similarity to cis-women than to cis-men with respect to body shape and body composition [34]. BMI was only slightly higher but the increase of obesity prevalence in transgender women was higher compared to cis-women. Thus, a subset of transgender women proved to be more prone for excessive weight gain [39]. In transgender men, body shape and body composition were within reference values for cis-women and cis-men. An earlier Tanner stage at start of treatment appeared to be associated with a closer resemblance of body shape to their affirmed sex [34]. The pre-treatment obesity prevalence was already higher compared to the general population, but the increase in prevalence was comparable to cis-men. For both transgender men and women, other cardiovascular risk factors such as fasting glucose, lipid profile, and blood pressure were similar or more favorable [39]. In addition, the psychological benefits of early medical intervention for young transgender adolescents have been established [30, 31]. One year after surgery, the GD was alleviated, psychological functioning had steadily improved, and well-being was similar to or better than same-age young adults from the general population [31].

## Summary

To meet the needs of youth with GI, a multidisciplinary team is required and therefore we recommend that children and adolescents are to be followed by an experienced multidisciplinary team with access to a well-trained team of mental health professionals, psychiatrists, endocrinologists, gynecologists, surgeons, and other healthcare providers. A phased trajectory is generally preferred and starts with psychological assessment, followed by medical interventions. Endocrine treatment consists of two phases: first the start of GnRHa to prevent the development of pubertal development (a fully reversible intervention) followed by the addition of with gender-affirming hormones, which leads to irreversible changes. Although many details and aspects of this approach are still unknown, it is of great importance that youth with GI are provided with care that improves their well-being. While taking steps in this process, the benefits and possible harms of each intervention should be carefully balanced.

## Abbreviations

BMD: Bone mineral density
GD: Gender dysphoria
GI: Gender incongruence
GnRha: GnRH analogues
IM: Intramuscularly
SC: Subcutaneously
PS: Pubertal suppression
TG: Transgender
